# Renal Oncocytoma: The Diagnostic Challenge to Unmask the Double of Renal Cancer

**DOI:** 10.3390/ijms23052603

**Published:** 2022-02-26

**Authors:** Francesco Trevisani, Matteo Floris, Roberto Minnei, Alessandra Cinque

**Affiliations:** 1 Urological Research Institute, San Raffaele Scientific Institute, 20132 Milan, Italy; francesco.trevisani@biorek.eu; 2 Unit of Urology, San Raffaele Scientific Institute, 20132 Milan, Italy; 3 Biorek S.r.l., San Raffaele Scientific Institute, 20132 Milan, Italy; 4 Nephrology, Dialysis and Transplantation, G. Brotzu Hospital, Università degli Studi di Cagliari, 09134 Cagliari, Italy; matteo.floris@aob.it (M.F.); rob.minnei@gmail.com (R.M.)

**Keywords:** oncocytoma, molecular profiling, renal function, molecular biomarkers, diagnosis, treatment, radical nephrectomy, partial nephrectomy

## Abstract

Renal oncocytoma represents the most common type of benign neoplasm that is an increasing concern for urologists, oncologists, and nephrologists due to its difficult differential diagnosis and frequent overtreatment. It displays a variable neoplastic parenchymal and stromal architecture, and the defining cellular element is a large polygonal, granular, eosinophilic, mitochondria-rich cell known as an oncocyte. The real challenge in the oncocytoma treatment algorithm is related to the misdiagnosis due to its resemblance, at an initial radiological assessment, to malignant renal cancers with a completely different prognosis and medical treatment. Unfortunately, percutaneous renal biopsy is not frequently performed due to the possible side effects related to the procedure. Therefore, the majority of oncocytoma are diagnosed after the surgical operation via partial or radical nephrectomy. For this reason, new reliable strategies to solve this issue are needed. In our review, we will discuss the clinical implications of renal oncocytoma in daily clinical practice with a particular focus on the medical diagnosis and treatment and on the potential of novel promising molecular biomarkers such as circulating microRNAs to distinguish between a benign and a malignant lesion.

## 1. Introduction

Renal oncocytoma (RO) is a benign renal neoplasm that is an increasing concern for urologists, oncologists, and nephrologists due to its difficult differential diagnosis from renal cell carcinoma (RCC) and frequent overtreatment. It displays a variable neoplastic parenchymal and stromal architecture, and the defining cellular element is a large polygonal, granular, eosinophilic, mitochondria-rich cell known as an oncocyte. Its history spans more than a century, as Schaffer was the first to describe oncocytes in 1897 [1], and in 1931, Hamperl observed their presence in several organs [2,3]. The first report of the presence of an oncocyte in renal tissue dates back to 1942, by Zippel [4], but it was not until 1976 that renal oncocytoma was described as a distinct clinicopathologic entity by Klein and Valensi [5].

## 2. Epidemiology and Risk Factors

### 2.1. Demographic Characteristics and Incidence

RO makes up a minority of renal masses, with a reported prevalence of 3–7.3% [6,7,8,9], but when considering only masses <4 cm or “small renal masses” (SRM), they may account for up to 18% of cases [10].

A retrospective study from Iceland reported a 0.3/100,000/year age-standardized incidence [8].

It usually presents in adults and older adults with 75% of patients in the 6th–8th decade of life [7], with a reported mean age ranging from 52 to 70.5 years across the literature [11,12]. A large contemporary study from the United Kingdom involving 1202 cases of RO reported a mean age of 66.8 years [13]. There is a male predominance, with a M:F ratio ranging from 1.25:1 to 3.3:1 [9,14].

### 2.2. Recurrence, Metastasis and Mortality

Oncocytoma is usually considered as a benign neoplasm due to its natural history and the extremely rare reports of metastasis and oncocytoma-related death. An older study reported a metastasis rate of 18%, with 5-year mortality of 65%; however, these results were biased by the retrospective design of the work. Furthermore, many of the analyzed cases predated the first series describing renal oncocytoma in 1976; consequently, it cannot be ruled out that foci of clear cell or pleomorphic cancer were present in unsampled areas of the specimen [15]. It is likely that many of these initial reports also included some cases of chromophobe RCC (chRCC) [16].

Several recent studies documented opposite findings, with no recurrence, metastasis, or RO-related mortality, during a mean follow-up duration ranging between 36 and 200 months across studies [8,17,18,19], including cases with pseudomalignant findings such as perinephric fat infiltration, or capsular invasion or renal vein invasion, without recurrences, lymph node infiltration or distant metastasis. One study reported a disease-specific mortality of 0% [20], while another found a 5-year overall survival of 63%, attributable mostly to other causes such as cardiovascular events or unrelated non-renal neoplasm, with no RO recurrence or metastasis [8].

Metastatic disease is extremely rare, and only scant data have documented systemic oncocytoma progression. Ordonez et al. reviewed 70 cases of oncocytomas and after a mean of 58 months of follow-up after diagnosis, two patients were in a metastatic stage, both had a secondary liver lesion, and one had liver and bone metastases and eventually died [9].

Metachronous renal cancer, such as clear cell renal cell cancer (ccRCC), may be present in some cases. Childs et al. reported their experience of 424 ROs, and 3% received a diagnosis of metachronous renal cancer at a median of 3 years, more frequently occurring in patients with a multifocal primary oncocytoma (H.R. 4.0; *p* = 0.007). However, RO does not seem to be associated with an increased risk for metachronous RCC [21].

Dechet et al. reported a significant association with concomitant RCC in 10% of cases, and a non-negligible incidence of metachronous oncocytoma was observed in 4% of patients during a post-nephrectomy follow-up of a mean duration of 41 months. Overall, oncocytomas were bilateral or multifocal in 5% and 6% of cases, respectively [22].

Sukov et al. observed that 38% of patients with a renal oncocytoma without cyclin D1 overexpression had multifocal renal lesions compared to 1% of patients with overexpressed cyclin D1; moreover, 32% and 0%, respectively, developed a subsequent oncocytoma after nephrectomy [23].

### 2.3. Risk Factor

To date, there are no established risk factors for sporadic RO, but there are well-documented associations between hereditary RO and genetic syndromes. Tuberous sclerosis complex (TSC) and Birt–Hogg–Dubé (BHD) syndrome are associated with an increased risk of oncocytomas, and it appears that the risk for multiple and bilateral disease is greater as compared to the general population [24,25].

Birt–Hogg–Dubé syndrome is an inherited autosomal dominant disease, characterized by underlying metabolic alterations caused by a loss of function of the folliculin/folliculin-interacting protein 1/folliculin-interacting protein 2 complex. Patients with BHD syndrome usually develop fibrofolliculomas and lung cysts and are at increased risk of renal cancer. Age at onset is <50 years, and presentation is multifocal or bilateral [26] and predominantly involves hybrid oncocytic/chromophobe tumors (HOCT) and chRCC. They may also be affected by ccRCC or papillary RCC (papRCC), and in 5% of cases, will develop an oncocytoma [27].

TSC is an inherited syndrome caused by a mutation in the TSC1 (hamartin) and TSC2 (tuberin) gene. One-third of individuals have autosomal dominant inheritance, while two-thirds are sporadic cases with de novo mutations that are predisposed to the development of skin lesions such as topical angiomyolipomas (AMLs), and renal, brain, heart, and liver manifestations, including neoplasms [28]. The most important renal manifestations are malignant AML and early RCC, particularly in subjects with the TSC2 mutation, which occur on average 20 years earlier than in the general population [29]. Oncocytomas are rare in TSC and carry an increased risk of developing bilateral, multifocal disease [30].

## 3. Classification of Renal Cancers and Histogenesis of Renal Oncocytomas

### 3.1. Classification

In the landscape of RCC, oncocytoma is considered as a benign neoplasm by the majority of experts [31], although some authors question this interpretation [32].

The 2016 World Health Organization (WHO) classification of tumors of the kidney identified various categories of renal cancer according to their histogenesis and age at onset, namely: renal cell tumors, metanephric tumors, nephroblastic and cystic tumors occurring mainly in children, mesenchymal tumors, mesenchymal tumors occurring mainly in children, mesenchymal tumors occurring mainly in adults, mixed epithelial and stromal tumor family, neuroendocrine tumors, miscellaneous tumors, and metastatic tumors. Oncocytomas are classified as “renal cell tumors”. The WHO classification grouped renal cancer according to a morphologic criterion (es. ccRCC, papRCC, chRCC, tubulocystic renal cell carcinoma), histogenesis and location (renal medullary carcinoma, collecting duct carcinomas), disease association (acquired cystic disease-associated RCC, hereditary leiomyomatosis and renal cell carcinoma-associated), genetic abnormality (MiT family translocation RCC, succinate dehydrogenase-deficient renal carcinoma) and finally unclassified renal cell carcinomas [33].

Several emerging entities, unreported in the last WHO classification, have been described in recent literature [34]; with regard to oncocytoma and its differential diagnosis, the most significant renal neoplasms include the high-grade oncocytic tumor (HOT) [35,36,37,38,39] and the low-grade oncocytic tumor (LOT) [40,41,42], as they lie within the eosinophilic spectrum of renal cell neoplasms.

Oncocytomas fall within a broader “spectrum” of renal neoplasms, the so-called eosinophilic tumors (including the eosinophilic variant of the chromophobe), characterized by predominantly granular and eosinophilic cytoplasm. Consequently, it has been, and to some extent, still is a field that is rife with misconceptions [19], controversies on its benign behavior [43], knowledge gaps, and in the last few decades, has passed through ever-changing classifications, evolving interpretations, and comprehension. At the same time, different emerging eosinophilic neoplastic renal entities have been described in the literature [44] (es. oncocytic papRCC, hybrid oncocytic/chromophobe tumors associated with BHD syndrome, renal oncocytosis, sporadic hybrid renal oncocytoma/chromophobe RCC) alongside other emerging histological entities not even acknowledged by the 2016 WHO classification (es. HOTs, LOTs, and eosinophilic solid and cystic renal cell carcinoma (ESC RCC) [34,45]).

While morphology is still fundamental for classifying such diseases, recent evidence and a deeper knowledge have led to a growing use of molecular characteristics, alongside immunohistochemical profiles and morphology. In 2021, the Genitourinary Pathology Society (GUPS) reviewed all this new evidence, post-dated with respect to the 2016 WHO classification, and introduced three categories of new entities on the basis of the supporting evidence: novel (different, independent validating studies), emerging (≥1–2 good independent studies, needing further validation) and provisional entities (few studies and limited data yet to be validated).

Among the meaningful new entities in the oncocytic renal neoplasm spectrum, novel entities (ESC RCC), emerging entities (eosinophilic vacuolated tumor (EVT) that replaced HOT and sporadic RCC with eosinophilic and vacuolated cytoplasm), and provisional entities (LOT) have been acknowledged [45]. Since most studies were carried out prior to the 2021 GUPS update, in this study, we will describe such neoplasms using the names they were assigned in their original paramount studies.

### 3.2. Histogenesis

Renal oncocytomas are considered as benign tumors arising from the intercalated type-A cells of the collecting duct. This hypothesis is supported by the expression of the collecting duct antigens in renal oncocytomas, such as the band 3 antigen [46], carbonic anhydrase C [47], the kidney-specific cadherin [48,49], the membrane-bound tyrosine kinase KIT [50,51], aquaporin 6 [52], beta-defensin 1 and claudin 7 [53], as well as the expression of biomarkers such as FOXI1, RHCG, LINC01187, identified by next generation sequencing techniques, shared with chRCC [6,16,52,54]. Nevertheless, a shared origin from a differentiated cell is debatable, considering the overall importance of the cancer stem cell theory in other RCC histotypes [55,56,57].

Oncocytomas exhibit not only several collecting duct markers (like parvalbumin) [58], but also an overexpression of mitochondrial-related and oxidative phosphorylation genes, similarly to the more aggressive chRCC, further suggesting that they may share a similar underlying biology [53,59].

However, despite a likely shared origin, chRCC and oncocytoma have antipodean prognosis and because of this must be distinguished, despite their sometimes-demanding differential diagnosis. In the meantime, our knowledge of their biology is rapidly growing and is shedding light on both their shared characteristics and distinguishing features. Several advanced genetic and omics studies (such as NGS studies [54], gene expression-exome-transcriptome and copy number alteration analysis [60], mass spectrometry proteome profiling [61]) have highlighted different tumoral-signaling pathway alterations, involving, above all, mTOR and c-erbB2 (dysregulated in chRCC) [16,52,62], and for small-cell variant RO, calcium-signaling pathways (PKCA, LCB4, PLCG2, members of CALM and ADCY families) [63].

## 4. Macroscopic and Microscopic Appearance

Renal oncocytomas usually display a defined macroscopic and microscopic appearance, immunohistochemistry (IHC) and cytological pattern, although diagnosis is sometimes challenging because of the presence of unusual or pseudomalignant histological features, the lack of defined diagnostic standard criteria in the differential diagnosis process, and above all, the presence of histologically similar entities lying in the eosinophilic spectrum of renal neoplasms [31].

### 4.1. Gross Anatomy

Macroscopically, the cut surface has a solid, not encapsulated, well circumscribed, brown (usually “mahogany brown”) or dark-red or tan-yellow appearance [6,9,64,65]; in renal oncocytomas, necrosis is usually not appreciated to the naked eye [19], but they can present hemorrhagic areas or cystic lesions in 20–27.5% of cases [9,66]. A central scar can be observed in 30–50% cases [9,65,66,67], but it is not a specific finding since it may be observed in other RCCs such as chromophobe and ccRCCs [67].

Chromophobe RCC has a gross anatomy that can sometimes mimic that of an RO on account of the solid, well-circumscribed lesion, with a homogeneous and brown cut surface, sometimes accompanied by a scar tissue band, although chRCCs are usually of a lighter brown, and a central scar is uncommonly observed. The mean dimensions are usually greater, with a reported mean of 8–8.8 cm [68,69].

### 4.2. Microscopic Anatomy

#### 4.2.1. Histopathologic Examination

Microscopically, the predominant or exclusive cellular parenchymal component is made up of oncocytes: large eosinophilic cells rich in mitochondria, with granular cytoplasm organized in different architectural patterns that sometimes coexist in the same sample and are surrounded by stroma [19,66].

The typical nuclear finding consists of round and regular nuclei containing prominent central nucleoli, with no significant mitotic activity and nuclear–nucleolar atypias [65,66,70]. The variably organized neoplastic cells are usually surrounded by, and sometimes dispersed in, an edematous myxoid or hyalinized stroma, which can also be observed in ccRCCs [65,66]. Moreover, in many cases (32–53%), oncocytomas display a typical but not diagnostic microscopic or macroscopic scar [19,66] that can also be found in RCCs with a slow growth and chRCC [67].

In the classic-nested pattern (about 50% of cases), neoplastic cells are arranged in nests or organoid structures surrounded by stroma, while the tubular and/or cystic pattern (6–29%) is characterized by dense areas of several dilated tubular structures similar to cysts or by actual cystic structures lined with a neoplastic eosinophilic epithelium. A mixed pattern sharing both the nest-organoid and the tubulocystic features can also be observed [19,66,71]. A solid pattern is a common finding (36–53%), sometimes concomitant with a nested appearance, but other less frequent patterns can still be commonly observed by the pathologist [71], such as a trabecular or papillary architecture [66,71], and should be considered together with cytologic, IHC and clinical features in the differential diagnosis [31].

#### 4.2.2. Histochemical and Immunohistochemical Staining 

Oncocytomas typically display overall scarce staining for cytokeratin 7 (CK7) [72,73,74,75], apart from small clusters of cells or individual CK7+ cells [76]. However, there is no widely accepted, clearly defined CK7-expression cut-off to distinguish an oncocytoma from other RCCs. One of the most often reported thresholds is <5% [31], although this is flawed by the focally increased expression in the central scar tissue [77].

Kidney-specific cadherin (Ksp-cad) typically stains ROs and chRCCs; thus, it can be used to differentiate these entities from other RCCs. Oncocytoma stains positively in 75–76% of cases, diffusely, and with a predominant membranous pattern, while chRCCs are stained in 86–92% of cases, similarly in a diffuse and membranous fashion [48,78], and their mean staining intensities were not found to be statistically different [78]. When analyzing whole histologic sections, both RO and chRCC stain positively in 100% of cases [48]. On the contrary, other RCCs, such as papillary or ccRCC, have significantly reduced [78] immunoreactivity for Ksp-cad (14–42% using tissue microarrays, 55–60% analyzing whole histologic sections) [48]. Mucinous tubular, spindle cell carcinomas, high-grade collecting duct carcinomas, renal medullary carcinoma and urothelial carcinomas show no immunoreactivity for Ksp-cad staining [48].

S100A1 is a recently introduced marker to which ROs are immunoreactive in 93–100% of cases, while chRCC stains negatively in 94–100% of cases [79,80]. According to other evidence, chRCC is non-immunoreactive in 75% of cases, and its eosinophilic variant is not immunoreactive in two-thirds of cases [81]. Other RCCs show frequent positive staining with S100A1, as has been observed in 94% of papRCCs and in 88% of high-grade and 52% of low-grade ccRCCs [80].

The 2014 International Society of Urological Pathology (ISUP) consensus for best practices in immunohistochemistry recommends the use of IHC only in borderline cases and identifies CK7 as the best choice in this setting, even if the eosinophilic variant of chRCC has reduced CK7 staining compared to the classical chRCC. In summary, the consensus states that in the differential diagnosis between oncocytoma and the eosinophilic variant of chRCC, cytologic and architectural observations can be integrated with IHC, and especially with CK7. Other oncocytoma-specific markers are less utilized, and it could be helpful to use Ksp-cad, while S100A1 is considered as a potentially useful marker that still needs to be fully validated [82].

KIT (CD117) is a membrane-bound tyrosine kinase that stains positive in >90% of RO or chRCC (100% and 96%, respectively); on the contrary, AML, papRCC, and ccRCCs are infrequently immunoreactive (17%, 5%, and 3%, respectively) for KIT [83].

RO and chRCC usually stains negative for Vimentin, although a focal positivity can be sometimes observed in the central scar of RO, while non-chromophobe RCC are usually immunoreactive. Vimentin and KIT are frequently used for the differential diagnosis between RO, chRCCs and non-chromophobe RCC because of their opposite IHC pattern [31].

Colloidal iron staining usually leads to negative staining in ROs, although in some cases, to circumscribed luminal [84] or focal cytoplasmic staining, with different patterns (perimembranous, apical or perinuclear) [85]. Conversely, chRCCs usually stain positive, with diffuse reticular intense staining [68,84], while ccRCCs display a focal, coarse, droplet-looking pattern, such as the eosinophilic variant of papRCC, although the latter finding may be a false positive due to the concomitant staining with Prussian blue performed in the study [84].

#### 4.2.3. Genetic Evaluation

In the routine clinical differential diagnosis of a renal cell carcinoma suspected of being an oncocytoma, genetic studies such as karyotyping, comparative genomic hybridization and fluorescence in situ hybridization could sometimes be helpful [70].

Karyotyping can be helpful when it is integrated with morphological and IHC findings, above all if cytogenetic findings show a diploid karyotype, loss of chromosome 1 or Y and, above all, rearrangement of 11q13 also involving the cyclin D 1 gene CCND1 [86]. On the contrary, the presence of other genetic abnormalities, such as loss of different chromosomes (es. loss of chromosomes 1, 2, 6, 10 and 17), strengthens the hypothesis of either a hybrid diagnosis or of an eosinophil variant of chRCC [31,86]. Another significant finding is that cyclin D1 positive staining of tumoral cells using IHC is associated with a CCND1 rearrangement detected by FISH [23].

It was recently proposed that oncocytomas could be subdivided into two types: type 1, displaying a diploid karyotype and CCND1 rearrangement, and type 2, exhibiting instead loss of certain chromosomes (namely, 1, Y but also X, 14 and 21). This led the authors to hypothesize that the latter represents a precursor to an eosinophilic variant of chRCC due to its overlap with the chromophobe histotype [87].

#### 4.2.4. Molecular Profiling

Molecular profiling of oncocytomas highlighted two different subtypes that share frequent inactivating mutations in mitochondrial genes encoded by both nuclear and mitochondrial genomes [88]. Mitochondria are crucial for the homeostasis of eukaryotic cells. Their expression is mainly regulated by a transcription program of biogenesis, whereas the withdrawal of the altered mitochondria is mediated through mitophagy, a selective type of autophagy [89]. Therefore, mitochondrial impairment may alter the respiratory chain with an accumulation of defective mitochondria, [90], thus suggesting a link between chronic metabolic deficiency and autophagy suppression. The presence of dysfunctional mitochondria in oncocytomas promotes energy crisis, AMPK activation, disruption of Golgi, lysosome autophagic cargo degradation and loss of mTOR signaling [87].

### 4.3. Grading

The Fuhrman grading system (Table 1) assesses nuclear size, nuclear pleomorphism and nucleolar prominence. It had been traditionally used to grade renal cell carcinoma due to its significant prognostic value but is flawed by suboptimal reproducibility when evaluating the worrisome morphological features of RCC [91,92,93,94]. Moreover, its prognostic significance for tumors other than ccRCC, such as papRCC or chRCC is less clear [95,96,97].

In 2012, another four-tier RCC grading system was presented by the ISUP, based on the highest grade of abnormality observed (nucleoli prominence, nuclear pleomorphism, presence of tumor giant cells or sarcomatoid and/or rhabdoid differentiation) [99], which is more reproducible than Fuhrman’s grade but whose prognostic value has been proven only for ccRCC and papRCC, but not for others such as chRCC [94,100,101,102] (Table 2).

The 2019 European Society for Medical Oncology (ESMO) and the 2021 European Association of Urology (EAU) guidelines on RCC support the use of the 2012 ISUP grading system over the Fuhrman grading system [105,106].

A large study of 93 oncocytomas in 80 patients highlighted that, even if a significant proportion of patients displayed atypical pathological features, no recurrence, metastasis or death was observed after a mean follow-up of 7.6 y, leading the authors to the peremptory conclusion that oncocytoma is a benign lesion that does not need a nuclear grading scheme. Consequently, a diagnosis of oncocytoma is still possible even if atypical findings are observed, since 42.5% of cases displayed prominent nucleoli (as in Fuhrman’s grade III-IV renal cell cancer), 50% had pleomorphism, and in 12.5%, there were focal areas of bizarre cells, 11.3% had perinephric fat infiltration, 10% showed renal parenchymal invasion without desmoplastic reaction, and 31.3% had hemorrhage [19].

### 4.4. Pseudomalignant Features and Histopathologic Differences between Eosinophilic Renal Neoplasms

#### 4.4.1. Pseudomalignant and Worrisome Features

The pathologist may observe irregular areas of nuclear atypia in an RO sample, namely, large nuclei, infrequent bi-nucleated or multinucleated cells, irregular nuclear margins, nuclear wrinkling, smudged chromatin, or infrequent intranuclear cytoplasmic invaginations [66,70]. Even in the presence of these findings, a diagnosis of oncocytoma should not be excluded if the clinicopathological context still supports its diagnosis [66]. There are some unappropriated cytologic features that lead us to consider other types of eosinophilic renal neoplasms, such as excessive mitotic activity. When more than one mitotic figure is observed, oncocytoma is usually excluded, while with one mitotic figure, its diagnosis may still be appropriate [70]. Trpkov et al. reported that in the 108 RO cases reviewed, 1.8% displayed rare mitotic figures and another 1.8% showed foci of coagulative necrosis. Clear cell foci may be found in 14.7% of cases, particularly in the central scar. Foci with chromophobe RCC-looking features limited to <5% of the tumor extension may be present [66], and in some cases, foci of small cells with scarce cytoplasm and with pseudorosettes may be observed, featuring an oncocytoma-like IHC and molecular profile [107].

Oncocytoma classically has a nested, solid and/or tubular architectural organization, but the observation of other patterns is not infrequent, as in the case of a cystic appearance with small endoluminal-protruding papillary formations and a trabecular pattern [19,66,71], and should be considered together with cytologic, IHC and clinical features in the differential diagnosis [31]. Besides increased mitotic activity, other findings usually rule out benign lesions such as oncocytoma, such as significant areas of clear neoplastic cells, as is observed in ccRCC, or significant necrosis or papillary structures, suggestive of other RCC subtypes [19].

Omiyale and Carton reported that 12.6% of the 159 cases of resected oncocytomas exhibited vascular invasion (*n* = 7), perinephric fat infiltration (*n* = 10) or both (*n* = 3), without recurrence, metastasis, or oncocytoma-related death over a mean follow-up of 25.6 months [108]. Wobker et al. reported different findings in their large multicenter study of 1474 cases, with only 1.5% of cases displaying vascular invasion, and a 2.5-year survival of 94.7% after diagnosis, without recurrence or metastasis, concluding that vascular invasion per se does not worsen the optimal prognosis of a confirmed diagnosis of an otherwise typical oncocytoma [109]. Amin et al. reported a morphologic re-examination of 80 cases of oncocytoma, with a non-negligible frequency of abnormal findings: in 42.5% of cases, they observed prominent nucleoli (similar to a ≥3 Fuhrman’s grade lesion, as used in the grading of malignant RCC), 50% displayed nuclear pleomorphism and even bizarre cells in 12.5%, while 11% showed perinephric fat infiltration, and 10% had renal parenchymal invasion, which instead would have been expected from a malignant neoplasm such as chRCC [19].

Luo et al. reported a case of oncocytoma displaying lymphovascular invasion with concomitant prominent intracytoplasmic vacuole-like spaces, which is seldom documented in the literature but does not preclude a proper oncocytoma diagnosis if the cytological, architectural and IHC findings are suggestive of oncocytoma, and its mimics have been excluded, particularly succinate–dehydrogenase-deficient renal cell cancer [110].

#### 4.4.2. Chromophobe Renal Cell Carcinoma

ChRCCs are well-circumscribed tumors that are usually bigger than Ros, and in one-third of samples, they may have a central scar [69,111]. They can present with different histologic architectures, leading to different subtypes. The characterizing tumoral cells are of two distinct morphologies: in classic chRCC, the main cellular element is a large cell with clear cytoplasm and a plant-like appearance, while in the eosinophilic variant of chRCC, the characterizing element is a smaller cell type with an eosinophilic cytoplasm.

Tumor cells are organized in solid cellular sheets or in nests or alveoli, with distinct or accentuated cellular margins, and they display a perinuclear halo with frequent irregular nuclei (“rasinoid”) [69,112]. Several other subtypes of chRCCs exist, but their characterization is beyond the scope of this review.

The nuclear findings may be similar to those of RO or may feature atypical findings, with frequent binucleations, a wrinkled nuclear membrane, and variable nucleolar features, and furthermore, because of the perinuclear and cytoplasmic clearing, there can be a finding of so-called “pseudo-koilocytc” atypia [69,112].

IHC can be helpful as an additional and integrative tool in the differential diagnosis between oncocytomas and other RCCs, since the former are usually characterized by a <5% CK7 expression and negative or luminal staining with colloidal iron, while an eosinophilic variant of the chRCC will usually stain positive for CK7 in >5% of tumor cells [70], although sometimes it may display scarce CK7 staining [82], and it will also show diffuse, reticular cytoplasmic staining for colloidal iron [84,85], which has led some experts to consider it as the best marker in this differential diagnosis context, although it is not formally an IHC marker [82]. S100A1 is a useful marker since most oncocytomas exhibit nuclear and cytoplasmic staining, while chRCCs are usually negative [80,81]. Ksp-cad will differentially stain oncocytomas with a cytoplasmic pattern, while chRCCs will show a mixed cytoplasmic-membranous pattern [48,78,113].

#### 4.4.3. High-Grade Oncocytic Tumors

HOTs are emerging entities, unrecognized by the 2016 WHO classification. Known also as “sporadic RCC with eosinophilic and vacuolated cytoplasm” EVT [45], they are characterized by the predominant presence of eosinophilic cells with granular cytoplasm, featuring vacuoles or eosinophilic inclusions, exhibiting mostly prominent and large nucleoli (similar to an ISUP grade 3 RCC). Besides the latter finding, no significant nuclear atypia were observed if not in few cases, with irregular nuclei and binucleation. HOTs usually appear as a solid brown single mass, while microscopically, they present a solid-nested architecture, sometimes tubulocystic or trabecular, such as an RO.

They stain positively with several markers (CK18, PAX8, SDHB, AE1/AE3) and negatively with TFE3, HMB45 and Melan-A. Similar to RO and chRCC, they are positive with KIT staining (64%) and negative for vimentin; furthermore, only sparse single cells are CK7+. Unlike chRCC, there are no multiple chromosomal losses, but there is loss of chromosome 1 and gain of chromosome 5q, with diploid karyotype [37,38]. HOTs are characterized by mutations in TSC1, or TSC2 or MTOR genes of the mTOR pathway [38,62].

He et al. were the first to document HOT and believed that this entity did not fit the criteria for RO, chRCC or for hybrid oncocytoma/chromophobe renal cell carcinoma (HOCT), because of the presence of prominent cytoplasmic membranes, cytoplasmic inclusions, thick-walled vessels, and large nuclei with prominent nucleoli (high-grade atypia) [37].

#### 4.4.4. Low-Grade Oncocytic Tumor

LOTs are characterized by solid, well-circumscribed, single, brown tumors, with a microscopic solid, nested architecture, of which is only focally tubular. They feature “oncotypic”, polygonal eosinophilic cells with vacuoles and inclusions, regular and round nuclei without significant nuclear atypia indicative of a low-grade, and fine chromatin. Tumoral cells were loosely organized and accompanied by myxoid stroma [40,114,115]. While HOTs are cathepsin K+, KIT+ and CK7− (only single cells stains positive) tumors, LOTs display a typical KIT negative/CK7 positive IHC pattern [115]. IHC analysis also showed immunoreactivity for PAX8, AE1/AE3, and e-cadherin, while negative staining was observed using HMB45, Melan A, CA9, CK20, CK5/6. Vimentin was negative in a study performed by Trpkov et al. [40], but it was positive in 35% of cases, although often only focally in a recent study by Akgul et al. [20]. They are usually diploid, in 2/9 cases, a disomic finding was observed, and del(19p13.3), del(1p36.33), and del(19q13.11) have been observed [40].

#### 4.4.5. Hybrid Oncocytoma/Chromophobe Renal Cell Tumor

Hybrid oncocytoma/chromophobe renal cell tumor (HOCT) is an entity acknowledged in the 2013 ISUP/Vancouver classification of renal neoplasias [103]. HOCTs have architectural and cytological features similar to both RO and chRCC. They exhibit a solid alveolar architecture [36], and they feature granular eosinophilic oncocytes with round nuclei, prominent nucleoli, indistinct cytoplasmic margins and tumoral cells such as those of chRCC, with slight eosinophilia, irregular nucleus, and distinct cytoplasmic borders [35]. Perinuclear halos and binucleated cells may be seen, but unlike chRCC, there are no raisinoid or irregular nuclei [36]. They stain positively for CK7 in the majority of cases, although only focally and negatively for vimentin [116], while they often display focal positivity for KIT [36]. They are immunoreactive for AEI/AE3, E-cadherin, and epithelial membrane antigen, while they stain negatively for racemase, CK20, CD10, carboanhydrase IX [36].

#### 4.4.6. Eosinophilic Solid and Cystic RCC

ESC RCCs are well-delineated tumors, with a defined capsule and the copresence of macrocystic and solid features [117,118]. Histopathological examination reveals a solid component with a diffuse nested or acinar architecture, with eosinophilic cells, basophilic coarse granules (“stippling”), round-oval nuclei, prominent nucleoli, and only focal nuclear membrane irregularities. The cystic component is characterized by trabeculae, and the cysts’ lumen is lined with neoplastic epithelium having a hobnail arrangement [117,118]. A common finding is the presence of histiocytes and lymphocytes. IHC reveals positivity for CK20, vimentin, PAX8, and AE1/AE3, with negative staining for CK7 and KIT, HMB45 and melan A [117,118]. They feature a bi-allelic loss or mutation of TSC1 and TSC2 [119], while HOT have non-overlapping mutations in these genes.

#### 4.4.7. Other RCCs

To differentiate an oncocytoma and a chRCC from other RCC histotypes, the pathologist can use markers such as KIT (CD117) and vimentin. KIT stains RO and chRCC positively, and vimentin leads to negative staining, while other RCC subtypes will result in positive stains with vimentin and negative stains with KIT [75,82].

A summary of the main histological and IHC features in RO differential diagnosis, with respect to chRCC, non-chromophobe RCCs, HOT, LOT, HOCT, ESC RCC is provided in Table 3.

## 5. Diagnosis and Staging

### 5.1. Diagnostic Approach

Renal oncocytoma diagnosis is incidental in 70–80% of cases brought to the clinician’s attention as a new renal lesion detected by imaging techniques [7,9] while only in a minority of cases are they diagnosed with imaging and pathological investigation following the onset of flank pain, clinical mass, hematuria, fever, and weight loss [9]. Renal oncocytoma accounts for a minority of renal masses, with a reported prevalence of 3–7.3% [6,7,8,9], but when considering only those <4 cm (SRM), they may account for up to 18% of cases [10].

The 2017 American Urological Association and 2019 ESMO clinical practice guidelines recommend the prompt use of laboratory examinations to study a suspicious RCC and suggest performing the following tests: serum creatinine, estimated glomerular filtration rate using the MDRD or CKD-EPI equation, complete blood count, lymphocyte to neutrophil ratio, lactate dehydrogenase, C-reactive protein, serum-corrected calcium, urinalysis by dipstick and microscopic evaluation to detect proteinuria, hematuria, pyuria and other urinary abnormalities [105,120]. This review will focus on the role of US, CT, MRI, SPECT and PET in the evaluation of solid renal masses, as well as on the suggestive features of RO and the distinguishing features with respect to other renal masses (Table 4).

#### 5.1.1. Diagnostic Role of Renal Ultrasound

Ultrasonography (US) is the backbone of the evaluation of renal masses alongside CT and MRI, but its diagnostic performance with solid neoplasms is not optimal since it may be difficult to differentiate a solid benign lesion, such as oncocytoma, from a malignant solid lesion relying solely on US evaluation [121]. Furthermore, its diagnostic accuracy is further reduced for smaller lesions, with detection rates of 21%, 28% and 58% for renal lesions of 5–10, 10–15 and 15–20 mm in diameter, respectively [122]. Therefore, about 3.7% of renal masses managed by nephrectomy are ultimately renal mass biopsy (RMB)-proven RO, according to a recent large UK nephrectomy registry analysis [13].

When a well-defined solid lesion without a fat component is identified, RCC, RO and fat-poor AML should be suspected, but a diagnosis of adenoma, leiomyoma, lymphoma, or metastases is still possible [123]. ROs are well-marginated, nodular, singular, or multiple homogeneous cortical masses, with variable echogenicity and often hypoechoic, isoechoic or mildly hyperechoic to renal parenchyma, especially when there is a higher percentage of stroma or when bilateral [124,125].

When present, a central stellate scar and a spoke-wheel vascular pattern may be observed [124], and color doppler shows that about 20% of lesions display mild vascularization [125]. Schwarze et al. found that 85% of cases were hyperenhancing, and half of them had delayed venous wash-out, while 8% were not hyperenhancing in the early arterial phase and had normal venous wash-out. Finally, the authors could not identify a specific microperfusion pattern [125].

Although many researchers claim good diagnostic accuracy of contrast-enhanced US (CEUS) based on the enhancing pattern observed, in the clinical setting, it is not a reliable differential diagnostic tool because of its diagnostic performance [126]. This could lead to a significant probability of misdiagnosis and/or mistreatment, which might result in an initial diagnosis of malignant neoplasm and subsequent nephrectomy for lesions that, only at the pathological examination of the surgical sample, will be diagnosed as benign lesions, such as oncocytomas and cystic lesions (es. In Bosniak category 3 cyst) [127].

#### 5.1.2. Diagnostic Role of Abdominal Computerized Tomography

Regardless of whether it is a serendipitous finding or prompted by clinical suspicion, the observation of a renal mass is not infrequent in a radiology unit, and the first step is to differentiate cystic from solid lesions; second, if the mass is not a cystic one, it must be determined by whether it contains areas of fat tissue, as is the case in cccRCC, or not, since in this case the main differential diagnosis will include fat-poor AML, RCC and oncocytoma [123].

Davidson et al. reported their experience with CT findings of 53 oncocytomas and 60 “renal adenocarcinomas” between 1980 and 1992, in which they evaluated a set of diagnostic criteria for these two entities in renal tumors bigger or smaller than 3 cm in diameter. Oncocytoma criteria included homogeneous attenuation and a sharp stellate central area of reduced attenuation, while the counterpart criteria for adenocarcinoma included the detection of any area of decreased contrast enhancement apart from the central area. These criteria were reported as insensitive and unspecific in differentiating oncocytomas and RCCs: 67% and 82% of >3 and <3 cm oncocytomas, respectively, met the oncocytoma criteria, while the remaining ones eventually met the adenocarcinoma criteria. On the contrary, 84% and 58% of >3 cm and <3 cm adenocarcinomas respected their predictive criteria, while the rest would have been classified as oncocytomas [128].

In a more recent study, Choudhary et al. retrospectively reappraised the CT findings of 21 histologically confirmed oncocytoma cases to evaluate the presence and the prevalence of classical CT findings, since they are considered hypervascular and homogeneously enhanced tumors, usually with a central stellate scar. However, after contrast media administration, two-thirds of cases were found to be isodense, and one-third were hypodense to the renal cortex. The typical stellate scar was identified both histologically and radiologically in only 10% of cases, while in 7%, CT was unable to detect a histologically proven scar; therefore, a total of 79% of oncocytomas did not exhibit a CT-appreciable stellate scar. The authors concluded that CT morphology alone was not reliable enough to distinguish oncocytoma from RCC, highlighting that the diagnostic standard was still histopathological examination [129].

Wildberger et al. reported the results of the examination of the CT scans of 65 RCC by seven diverse radiologists in a consensus conference also involving pathologists and urologists. Only 12% of RMB-proven oncocytomas were properly diagnosed [130].

In 2021, a retrospective study by Li et al. evaluated the diagnostic value of various CT-detected features in 25 patients with oncocytoma and in 73 with chRCC, all of which presented the classic stellate scar. A number of distinguishing findings positively correlated with the diagnosis of oncocytoma: right kidney location, hypodensity in non-contrast CT, segmental enhancement inversion (SEI) and thickening of the perirenal fascia [131].

Wu et al. conducted a comparative study between the CT features of 56 cases of oncocytoma and 54 of chRCC, using multidetector CT (MDCT). They observed that certain features, none of which were pathognomonic for oncocytoma, were observed more commonly in RO than in chRCCs and might be helpful in the differential diagnosis: the classic stellate scar, a spoke-wheel enhancement pattern, and lastly, SEI [132]. 

Scialpi et al. recently explored the role of triphasic MDCT in the differential diagnosis between RCC and oncocytoma, focusing their research on a qualitative and quantitative evaluation of the imaging features of ≤4 cm lesions, in the cortico-medullary, nephrographic and pyelographic phases. They reviewed 21 oncocytomas and 23 RCCs that were, respectively, hypervascular/hyperdense in 43% and 56% of cases, while pseudocapsule was only observed for RCCs, and a homogeneous appearance was exhibited by only 48% of oncocytomas. Hypervascular RCC did not show any statistically significant difference in mean attenuation values versus the renal cortex in the three phases, while on the contrary, oncocytomas did, especially in the nephrographic and pyelographic phases. With respect to the renal cortex, hypovascular oncocytoma and RCC displayed a statistically significant difference in all three post-contrast phases. The authors concluded that the absolute attenuation and the quantitative amount of enhancement were weak predictors for distinguishing the two different neoplasms. They rather considered triphasic MDCT as a significative imaging modality for patient with SRM, since it increases the diagnostic specificity, potentially influencing the therapeutic decision making between radical nephrectomy (RN) or partial nephrectomy (PN) [133].

Another study evaluating the role of MDCT for SRM by Ching et al. found that oncocytomas had greater attenuation and enhancement with respect to other RCCs, and even more, they a observed a different pattern of CT findings between oncocytomas and chRCC [134]. Bird et al. found a similar result in their study on four-phase CT in <4 cm oncocytomas and RCCs, since the former displayed a greater mean enhancement change in arterial, venous and delayed phase, the enhancement pattern was significantly different, and the mean relative contrast extraction at the end of the delayed phase was higher for oncocytomas. The authors stated that multiphase CT is a helpful tool in differentiating small oncocytomas from small RCCs, even describing a >500% and >50% threshold for the arterial phase enhancement and washout values, respectively, that are suggestive of oncocytomas [135].

Jung et al. described the use of biphasic MDCT and the detection of SEI in the characterization of small renal oncocytomas and their impact on therapeutic decision making. SEI is a radiological feature characterized by the presence of differently enhancing areas (“segments“) of the same lesion, whose enhancing pattern varies between post-contrast phases. ROs usually have two segments: in the corticomedullary phase, one is well enhanced and the other is less enhanced; and in the early excretory phase, they have the opposite enhancement pattern (the well-enhanced segment becomes less enhanced and vice versa). The authors assumed that this finding was suggestive of small oncocytomas. With this criterion, the MDCT had 80% sensitivity and 99% specificity in distinguishing <4 cm oncocytomas and RCCs, proving to be a helpful tool in differential diagnosis, thus suggesting it may directly influence the therapeutic approach to SRM [136]. Regardless, it is still controversial of whether SEI is a specific oncocytoma feature. O’Malley et al. found contradictory results in their experience, with 16 cases of oncocytomas, 15 RCCs and 15 controls, and with renal masses ≤4 cm in diameter, reappraised/reevaluated by three different reviewers. Two reviewers observed no SEI in any of the lesions, while the third only found it in 6% of oncocytomas [137]. These results were similar to those reported by McGahan et al., with only 6% of oncocytomas displaying SEI and with similar MDCT delay for image acquisition in the last enhanced phase [138], while Jung et al. showed a significantly longer delay in image acquisition [136], suggesting remarkable heterogeneity in methods as a possible explanation for their discordant findings [137].

A few older studies documented that even oncocytomas may display calcification, as do malignant RCCs, further highlighting how difficult the imaging differential diagnosis of renal masses can be [9,139,140].

The application of radiomics in the study of renal neoplasms has increasingly been reported due to its remarkable diagnostic potential and future role in personalized medicine. Digital images carry an overwhelming amount of information not accessible to the human eye. By high-throughput computing, a huge amount of quantitative data can be extracted from medical images, transformed into data, stored in a system of independent databases working as a single entity (“federated databases”), and statistically analyzed with different goals in mind, such as supporting clinical decision making and predicting underlying histologic features [141]. Few studies have explored radiomics in the characterization and differentiation of oncocytomas from other RCCs. Moreover, while extremely promising, this technology comes with pitfalls, such as suboptimal reproducibility of findings because of a problem in image acquisition, data processing and statistical model building, eventually leading to validation issues [142]. In a recent retrospective study, Li et al. built and validated three predictive models, specifically in an effort to distinguish between oncocytomas and chRCCs with a central scar. The models were based on three-phase-enhanced CT, and their study included a clinical model, radiomics signature, and a radiomics nomogram. Forty-seven cases of oncocytomas and 94 of chRCCs were included, 10 clinical factors were used in the study (including SEI) and 2553 radiomics features were extracted. The best performing of the three was the validated radiomics nomogram, which integrated SEI of the lesions and radiomics signature, displaying an AUC of 0.988, sensitivity of 89.5%, specificity of 97.4% and accuracy of 94.7%, outperforming the other models and showing promising pre-operative and predictive performance in a selected clinical scenario: distinguishing two sometimes indistinguishable renal tumors. These results also need to be tested and validated on clinical grounds [143].

#### 5.1.3. Diagnostic Role of Abdominal Magnetic Resonance Imaging

Several studies have documented that conventional morphological imaging does not discriminate between benign and malignant solid renal lesions [144,145]. RCC usually has a T-1 weighted isointense heterogeneous signal, and because of the possibility of concomitant necrosis and/or hemorrhages [146], the T-2 weighted image findings correlate with the RCC subtype since ccRCC usually displays T2 hyperintensity while papRCC shows T2 hypointensity [147,148], sometimes harboring evident hemorrhagic or necrotic areas responsible for their heterogeneity [149]. Conversely, oncocytomas are usually described as having T1-weighted hypointensity and T2-weighted hyperintensity [149], although it is known that they possess variable and nonspecific MRI appearance and a significant overlap with other RCC imaging features, all of which lead to a complicated preoperative differential diagnosis [146].

Pretorius et al. found an impactful overlap between oncocytoma and RCC MRI appearance: oncocytoma had homogeneous or heterogeneous signal intensity in 40% and 60% of cases, respectively. Forty percent displayed isointensity and 60% were hypointense in T1 sequences, while in T2-weighted images, 40% displayed hyperintensity, and 40% showed a hypointense signal. With regard to contrast enhancement, 60% of oncocytomas were isointense in the dynamic post-gadolinium phase, and 40% showed a hypointense signal, while in the delayed phase, the proportion shifted to 80% and 20%, respectively [150]. The central scar, which is occasionally observed in some RCCs, including oncocytomas [149], exhibits a hypointense signal in T1- and hyperintensity in T2-weighted sequences [147]. Nonetheless, without gadolinium administration, it can still be mistaken for a central necrotic area that has the same radiological appearance that is typical of a malignant tumor [147]. Contrast MRI should be performed since the central scar may exhibit delayed enhancement, while a necrotic area will not [151], and could exhibit a stellate or spoke-wheel enhancement pattern [146].

Taouli et al. hypothesized that diffusion-weighted imaging (DWI) could be more informative and even an alternative to contrast-enhanced MRI. In their work, they observed that combined DWI and contrast-enhanced imaging led to 100% sensitivity and 96% specificity in the characterization of renal masses. Regarding renal oncocytomas, they observed that these tumors display an apparent diffusion coefficient (ADC) significantly higher than that of other solid RCCs. When a fat-containing solid and enhancing renal lesion was excluded, sensitivity and specificity for the differential diagnosis between oncocytoma and RCC were respectively 90% and 83% by combining ADC information to contrast-enhanced MR imaging [152]. De Silva et al. recently evaluated DWI MRI imaging and ADC ratio for differentiating benign from malignant renal lesions, in a study that featured a 3T MRI. They observed that renal oncocytomas have a significantly higher ADC value, a mean of 2.16, compared to other masses, with ccRCCs having the second highest ADC of 1.5 mean, therefore demonstrating a statistically significant difference between this malignant histotype and non-clear cell RCC. Thus, DWI/ADC MRI is a helpful means in the differential diagnosis between oncocytoma, AML and malignant RCC; moreover, it may help in discriminating RCC histotypes in cases where RMB or surgery are contraindicated [153]. Another study found a similar ADC between oncocytoma and ccRCC, and thus differential diagnosis may not be possible by relying on this parameter alone [146,154].

Another oncocytoma-RCC overlapping feature is known as segmental enhancement inversion (SEI), an imaging finding that can be observed after gadolinium administration, characterized by a pattern variation in the mass heterogeneous enhancement [146]. Rosenkrantz et al. [145] and later Schieda et al. [155] highlighted that SEI is not useful in oncocytoma diagnosis or in the differential diagnosis with chRCC. As Bird et al. reported, oncocytomas display a significantly high washout value of contrast media at the end of the delayed phase [135], higher than other RCCs such as ccRCC, chRCC or papRCC, the cut-off being >50% of administered contrast media undergoing wash-out [146].

Oncocytomas and chRCCs not only share a similar histogenesis and some histological features, but also have a significant overlapping imaging appearance. They may present as well-defined, homogenous solid lesions, and they may harbor a central stellate scar with a spoke-wheel enhancement pattern [145,146,156]. However, the absence of definitive MRI features prompts a discussion between the radiologist and the urologist regarding further work-up and management, since surgical resection, RMB or follow-up imaging can all be the right decision depending on the patient [146].

Some recent studies on the application of multiparametric MRI in the characterization of renal masses have provided interesting results. Cornelis et al. retrospectively analyzed 100 consecutive pathologically proven solid renal tumors lacking a macroscopic lipid component, 16 of which were actually oncocytomas, and reviewed a wide array of MRI parameters, namely: double-echo chemical shift, dynamic contrast-enhanced T1- and T2-weighted images, ADC mapping, signal intensity index, tumor-to-spleen SI ratio, ADR ratio, and wash-in and wash-out indices between phases. Regarding oncocytoma, a surprising 100% and 94.2% specificity was found in differentiating it from chRCC and ccRCC, respectively [157]. Lanzman et al. studied the application of arterial spin-labeling perfusion MRI in the evaluation of solid renal masses, finding that different perfusion levels correlated with different respective RCC histotypes, eventually observing that oncocytomas have higher perfusion than other RCCs [158].

Zhang et al. investigated the potential usefulness of desorption electrospray ionization (DESI)-mass spectrometry imaging in differentiating renal oncocytomas from RCCs and between RCC subtypes in 71 patients, obtaining 73,992 mass spectra to be used in a predictive model based on the least absolute shrinkage and selection operator (Lasso). They reported a remarkable 100% accuracy in distinguishing renal oncocytomas from chRCCs, and an overall 100% accuracy per patient in the prediction of normal parenchyma, renal oncocytoma and RCC in the studied sampled tissue [159].

Razik et al. recently studied the application of radiomics, namely the role of magnetic resonance texture analysis (TA), in the differential diagnosis between RCC (*n* = 34), oncocytoma (*n* = 6) and lipid-poor AML (*n* = 14), based on 1.5 T MRI images. TA allows for a quantitative analysis of a given medical image focusing on its heterogeneity, with respect to the greyscale. Six parameters were identified and displayed promising diagnostic performance with an AUC > 0.8 in discriminating RCCs from lipid-poor AMLs and oncocytomas [160]. As observed for the MDCT-based radiomics technique cited elsewhere in this review [142], these results need to be further reproduced and validated in larger studies featuring increased homogeneity in the different phases of radiomics analysis [160].

#### 5.1.4. Diagnostic Role of Renal SPECT Scan

Gormley et al. studied the application of 99mTc-sestamibi in the noninvasive diagnosis of renal masses in six patients using a planar acquisition technique, with the aim of distinguishing renal oncocytomas from other RCCs based on their overexpression of mitochondria and sestamibi retention by mitochondria-rich cells, the only oncocytoma that displayed increased uptake with respect to other tumors (including 3 RCCs) [161]. Rowe et al. evaluated the role of SPECT/CT with 99mTc-MIBI in distinguishing renal oncocytomas from other renal masses, in an initial experience including six patients [162] and in a subsequent prospective study of 50 patients presenting with a cT1-staged renal mass in the preoperative setting [163]. In their first experience, they documented similar increased uptake of 99mTc-sestamibi with respect to the normal parenchyma, while other RCCs displayed consistent, reduced uptake [162]. In their subsequent work, the preoperative imaging findings were compared to the post-surgical histopathological diagnosis. Of the six oncocytomas, 83.3% had been properly diagnosed, as had the two HOCT, with two false-positives, thus resulting in global sensitivity of 87.5% and specificity of 95.2% in diagnosing oncocytoma and HOCT [163].

Another team explored the role of 99mTc-sestamibi SPECT/CT, corroborating the aforementioned results, with 91.6% sensitivity in oncocytoma detection, 3/3 hybrid oncocytoma/chromophobe RCCs resulting as positive, and 91.7% of RCCs as negative (one papRCC was mildly hypercaptant) [164]. Zhu et al. recently investigated the role of dual-phase 99mTc-MIBI SPECT/CT (image acquisition 30 and 90 min after radiotracer administration) in 148 cases of RMB-proven solid renal tumors, in characterizing their enhancing features. Oncocytoma represented 2.7% of cases, and lipid-poor AML made up 5.4%; among them, 91.9% were malignant. The benign lesions exhibited a higher early (ERUV) and delayed relative uptake value (DRUV) with respect to the malignant lesions (*p* < 0.0001). With a cutoff of 0.53 for ERUV and 0.5 for DRUV, sensitivity in distinguishing them from malignant neoplasms was 100%, while specificity was 96.3% and 96.6%, respectively. Moreover, the delayed phase imaging appeared to be more diagnostically accurate, leading the authors to speculate that improved accuracy could be obtained from image acquisition at ≥120 min after radiotracer administration [165].

#### 5.1.5. Diagnostic Role of Renal PET

The first reported case of a hypermetabolic oncocytoma detected by 18F-FDG PET scan was an unexpected finding in a comparative PET vs. CT study on characterizing and staging renal cancers, although the intensity of its uptake was not quantified [166]. Blake et al. documented a case of renal oncocytoma studied with 18F-FDG PET/CT that displayed a 4.4 cm enhancing mass with slow interval growth and intense radiotracer uptake [167]. Makis et al. reported a similar case of multiple and bilateral hypercaptant oncocytomas, supporting the belief that an FDG-avid renal lesion alone does not rule out an oncocytoma [168]. On the contrary, although PET/CT is not the standard diagnostic modality for evaluating a primary renal mass, (malignant RCCs display variable FDG-avidity) [168], its diagnostic sensitivity for primary RCC is low, at 60%, while specificity is 100% [169]. Shirki et al. reported an incidental oncocytoma diagnosis in a patient being evaluated for metastatic prostate cancer by 11C acetate PET scan and a renal hypercaptant lesion with respect to renal parenchyma, which was eventually diagnosed pathologically as an RO [170].

#### 5.1.6. Diagnostic Role of Renal Tumor Biopsy

Core needle biopsy (CNB) sampling has greater sensitivity compared to fine needle aspiration (FNA), 92–97% vs. 76–78%, respectively [171,172], and it is a more reliable diagnostic tool and allows for better characterization of RCC subtypes [171].

Several studies have explored the usefulness of a combined CNB and FNA approach. Yang et al. reported an increased diagnostic rate (94%) for the combined approach over FNA (72%) and CNB alone (87%), as well as greater sensitivity and diagnostic accuracy (92% and 94%, respectively) [171]. Barwari et al. similarly reported a relative increase in diagnostic accuracy of 3.5–14% [173].

The potential benefits of performing a renal mass biopsy (RMB) in SRM instead of upfront surgery with subsequent pathological examination include: avoiding overtreatment of benign lesions, distinguishing between a primary or a secondary renal neoplasm and influencing its treatment, and distinguishing primary RCC histotypes since they carry different prognosis and treatment [174]. The diagnostic accuracy of CNB is still a concern since diagnosis is inaccurate in one-third of cases, most likely because the sampling is incomplete and because of interobserver variability [175].

Richard et al. reported their 13-year single-center experience of 529 patients with a SRM (≤4 cm) who underwent at least 1 RMB. Since the first biopsy was not diagnostic in 10% of cases, a second one was performed with a diagnostic rate of 83%; consequently, 94% were overall diagnostic [176]. The positive predictive value (PPV) in characterizing a renal mass as malignant is more than 99%; on the contrary, the negative predictive value (NPV) is about 63%, with a remarkable proportion of false negative results, erroneously diagnosed as benign and eventually found to be malignant [177,178].

Maturen et al. reported their experience of 276 imaging-guided core kidney biopsies, in an effort to evaluate its impact on clinical management. They reported a PPV and an NPV of 100% for malignancies, with 97.7% sensitivity and 100% specificity, and in 60.5% of cases, RMB led to a significant change in the management of the lesion. A low complication rate confirms the safety of this imaging-guided procedure, with 0.7% of cases requiring a blood transfusion because of a hematoma and 0.7% exhibiting a delayed renal pseudoaneurysm [179].

Hoare et al. described their experience with 148 patients who underwent US- and CT-guided RMB for T1 and T2 renal lesions. They reported 96.2% sensitivity, 87.5% specificity, 98.7% PPV and 70% NPV, with a diagnostic accuracy of 90.7%. A second RMB was needed in 7.4% of cases (*n* = 11), and four cases that had been classified as benign were diagnosed as malignant. One hundred three cases (69.5%) were malignant, and in 58.1%, the pathologic and radiologic features led to nephrectomy. In this study, only 2% (*n* = 3) of cases had a complication of at least grade II Clavien: one case of subclinical and limited pneumothorax, one case of painful moderate perinephric hematoma, and finally a severe renal bleed that led to an emergency nephrectomy, inotrope administration and ICU transfer in a patient with a positive history for hypercoagulability and significant retroperitoneal hemorrhage [180].

Alle et al. reported remarkable findings in their review of 183 consecutive cases of patients who underwent percutaneous RMB with CT or US guidance. In the end, 169 patients and 184 RMBs were analyzed, and subsequent nephrectomy was performed in 28 cases (one case had insufficient tissue sampling). In all 27 cases, the benign and malignant diagnoses were confirmed, with 100% sensitivity, specificity, PPV and NPV, and in six cases, the surgical histopathology changed the initial RCC histotype diagnosis. They reported only one major complication involving a patient in end-stage kidney disease on hemodialysis who underwent RMB and concomitant radiofrequency ablation. A traumatic arteriovenous fistula with a retroperitoneal hematoma warranting transfusion support and arterial embolization were observed. Thirteen minor complications were documented, with 10 cases of hematoma that were conservatively managed, one case of episodic hematuria, one of vasovagal reaction and one case of abdominal discomfort [181].

According to a recent systematic review and meta-analysis, an overall mean 8.1% complication rate was observed for percutaneous RMB. A median of 4.3% and 0.7% cases developed Clavien 1 and 2 hematomas, respectively—the former requiring, by definition, a blood transfusion. Only one patient developed gross hematuria with urinary retention due to urinary blood clot, and another patient underwent endovascular embolization because of post-biopsy pseudoaneurysm formation. One case of RCC seeding was observed as was one case of septic shock. Finally, a 92% overall diagnostic rate was observed [182].

The 2019 ESMO guidelines on renal cell carcinoma support the usefulness of renal tumor CNB in confirming the malignant nature of a renal mass, recommending its use above all before treatment with ablative procedures (III, B) and before systemic treatment in the metastatic stage (III, B). Even more, in patients with local disease and a cortical renal lesion of ≤3 cm, performing an RMB for the pathological malignant and subtype diagnosis is recommended, above all in the following clinical scenarios in which local ablation with radiofrequency, microwave or cryoablation are therapeutic options for frail patients, patients at high surgical risk, those with a solitary kidney, and individuals with compromised renal function, multiple bilateral tumors or hereditary renal cell cancers. RMB is also recommended for patients with SRM managed by active surveillance (elderly patients with impactful comorbidities, short life expectancy and solid tumors <4 cm), since benign tumors are frequent in SRM (III) [105].

The 2017 AUA guidelines for renal mass and localized renal cancer state that an RMB should be considered for diagnostic purposes to enhance the renal mass in the following cases: when a lesion is suspected of being metastatic because of an infiltrating, poorly enhanced and multifocal imaging appearance and recent history of malignancy with potential renal metastasis (such as in lung, colon, thyroid cancer, melanoma and lymphoma); when an inflammatory or infectious disease is suspected on the basis of suggestive clinical manifestations and a history of prior diagnosis. Multiple CT or US-guided CNB (≥2–3) sampling is preferred over FNA in patients with a solid renal mass because of a higher diagnostic yield (moderate strength, grade C). On the contrary, the guidelines state that in certain scenarios, RMB is not required, and based on expert opinion, these scenarios include: young or healthy individuals who are concerned about the possibility of misdiagnosis related to the histopathologic examination and are unwilling to accept this risk; older or frail patients about to undergo a conservative treatment strategy, regardless of the eventual histopathological examination diagnosis [120].

### 5.2. Staging

The 2017 AUA and 2019 ESMO guidelines support the use of the TNM 8th edition [183] to systematically classify renal cancer stage [105,120]. The 2019 ESMO guidelines for the diagnosis, treatment and follow-up of RCC recommend (III, A) specific staging procedures, namely a contrast-enhanced CT of the chest, abdomen, and pelvis, and in case of allergy to the contrast media or renal insufficiency, a high-resolution chest CT without contrast administration, with an abdominal MRI. Bone scan and brain CT or MRI are not routinely recommended (III, A) unless there is a clinical suspicion or if prompted by abnormal laboratory results [105].

The 2017 American Urological Association guidelines on renal cancer, renal mass, and localized renal cancer recommend using high quality, multiphasic cross-sectional abdominal imaging such as MDCT or MRI whenever possible, with the administration of contrast media to characterize and stage the renal mass. However, chest imaging can be tailored depending on tumoral risk; chest radiography may be appropriate for lower risk tumors, while a chest CT can be reserved for patients with significant clinical manifestations or high tumor risk (thrombosis, adenopathy, tumor size, infiltrating features in diagnostic images, extensive necrosis) [120].

## 6. Treatment

Oncocytomas represent the most common surgically removed benign renal tumors, with an incidence ranging from 4% to 7% of all kidney surgeries for neoplasms [184]. This high rate of surgery for a non-malignant process is the result of the underuse of percutaneous renal surgery and from the inability of the current radiological techniques to distinguish between malignant and benign lesions with an acceptable degree of accuracy [184,185]. Moreover, in the urological scenario, clinicians are still divided on the interventional management of oncocytoma, with a high percentage of surgeons who prefer to operate rather than considering a period of surveillance [186]. This attitude derives historically from two precise reasons that warrant explanations. The first one is related to the fear of neglecting a malignant process which, from a radiological point of view, looks similar to a benign mass. It is intuitive that if a surgeon reassures a patient on the benign nature of an incidental mass that eventually proves to be malignant, the clinical and legal implications stemming from this medical error could dramatically change the patient’s lifespan and the physician’s career [187]. The second aspect, conversely, is attributable to the possible growth of the tumor over time, resulting in the development of symptoms and chronic kidney disease (CKD) due to the pressure of the lesion on the healthy renal parenchyma [188]. Therefore, previous evidence suggests that renal oncocytomas measuring more than 5 cm or increasing by 5 mm or more per year should be surgically treated. 

Radical nephrectomy (RN) or partial nephrectomy (PN) are the first lines of therapy for benign renal masses when the surgical approach is preferred to watchful waiting [189]. Both types of surgeries remain widespread in the management of already diagnosed oncocytomas (using RMB) and presumed renal cell carcinomas with a post-operative histological diagnosis of a benign lesion [190].

In the last decade, PN has been considered as the surgical approach of choice in the RCC panorama due to the lower rate of medical side effects compared to RN [191,192]. Nephron-sparing surgery and minimally invasive techniques have improved not only the surgical results but have also decreased the rate of mortality and morbidity related to RN [193]. In particular, PN displayed a significant improvement regarding functional aspects related to cardiovascular events and renal impairment, such as acute kidney injury (AKI) and CKD [194]. This aspect gained importance both in fragile and in healthy patients. In the former category of patients, the preservation of renal function over time and the reduced risk of end stage renal disease displayed had a huge impact on the lifespan of patients by limiting the effect of the concomitant comorbidities and resulting in a better quality of life [195]. At the same time, especially in young patients, the removal of an entire kidney due to a benign mass using RN not only compromises the possibility of these subjects to donate a kidney but it also leaves them with the fear of possibly developing CKD in the distant future [196]. Nevertheless, PN remains a type of surgery with substantial risks associated with surgical resection, with 20% of patients experiencing inpatient complications and with a 60-day mortality rate of 0.4% [197]. For this reason, especially in fragile elderly patients affected by several diseases, over the past years, the possibility of proposing active surveillance to monitor the growth of the renal mass has become increasingly more accepted because several studies have underlined that most oncocytomas slowly increase in size over time [198,199]. Moreover, the rationale that oncocytoma might decrease renal function if surgery is not performed remains controversial and debated in the literature due to the activation of compensatory hyperfiltration mechanisms in the healthy parenchyma. Finally, recent works have underlined that even PN shows a non-negligible risk of AKI and CKD over time [200,201].

Therefore, in accordance with the EAU, active surveillance may be a safe option for managing oncocytoma in appropriately selected patients, preferably with histological confirmation via percutaneous RMB [106].

## 7. MiRNAs as Diagnostic Biomarkers for Oncocytoma

The differential diagnosis of benign renal oncocytoma from malignant renal tumors and their eosinophilic or oncocytic variants is a clearly unmet clinical need. Therefore, there is an urgent demand for non-invasive and specific biomarkers for oncocytoma.

MicroRNAs (miRNAs) are a class of small non-coding RNAs that represents a promising non-invasive cancer biomarker tool [202,203,204,205,206,207,208,209]. MiRNAs regulate gene expression at the post-transcriptional level by pairing to the 3′ untranslated region (UTR) of target messenger RNAs (mRNAs) through translational repression or mRNA degradation [210,211]. This class of molecule is suitable as a non-invasive or minimally invasive biomarker because miRNAs are present in all biological fluids such as urine and blood (where they are abundant and stable) and can be quantified by highly sensitive, accurate, and reproducible measurement methods (such as quantitative polymerase chain reaction (qPCR)) [212,213,214]. In addition, it has been extensively demonstrated that their levels of expression could correlate with different pathological conditions in various medical fields, becoming excellent physiological parameters of both health and disease [215].

Their potential utility as predictive, diagnostic, and prognostic biomarkers in RCC patients was extensively described in previous reviews [207,208].

Here, we have focused on the potential ability of these molecules in the serum and urine samples of patients to discriminate renal oncocytoma from malignant renal masses, even if there are few data on this issue in the literature.

Von Brandenstein M. et al. found that miR-498, miR-183, miR-205, and miR-31 are suitable urinary biomarkers for the presurgical diagnosis of oncocytoma [216]. These miRNA levels were significantly higher in the urine of oncocytoma patients (*n* = 5) compared to those of ccRCC (*n* = 10), papRCC (*n* = 6), chRCC (*n* = 5), and urothelial carcinoma patients (*n* = 5) [216]. In addition, miR-183 and miR-498 urinary levels markedly decreased after tumor resection [216]. Particularly, miR-183 urinary levels dropped to about one-twentieth of presurgical levels in oncocytoma patients and remained basically unchanged in RCC and urothelial carcinoma patients, while miR-498 was no longer detectable in the urine of oncocytoma or RCC patients [216]. Furthermore, these miRNAs are involved in some pathways or mechanisms known to be specifically relevant for oncocytoma [216].

miR-498 is associated with the formation of vimentin 3, a spliced variant of Vimentin, ending with a unique C-terminal ending after exon 7, which differentiates it from the full-length version that has nine exons and a recognition site for miR-498 [216,217]. Vimentin 3 is overexpressed exclusively in oncocytoma and allows for IHC differentiation from malignant RCC [217].

miR-183 is upregulated in high CO_2_ levels [218] as occurs in oncocytomas due to their unique mitochondrial dysfunction caused by the absence of mitochondrial complex I [219]. In human alveolar carcinoma cells, increased amounts of CO_2_ are able to downregulate the enzyme isocitrate dehydrogenase 2 (IDH2) of the Krebs cycle [218]. Furthermore, it has been reported that high expression levels of miR-183 in various types of gliomas are associated with a downregulation of IDH2, which has complementary sequences to miR-183 in its 3′-UTR [220]. However, in contrast to expectations based on these studies, Von Brandenstein M. et al. observed strong IDH2 expression by immunohistology in oncocytomas and to a lesser degree in eosinophilic/oncocytic variants of chromophobe carcinoma [216].

Finally, miR-205 and miR-31 have been reported in the literature to downregulate protein kinase C (PKC) epsilon [221,222], which is downregulated in oncocytoma [223].

miR-205 was found to be deregulated in various adenocarcinomas [224,225,226,227,228,229,230,231,232,233,234].

Most noteworthy, miR-205 is downregulated in prostate cancer [235], and in most published works it was reportedly downregulated also in bladder cancer [236,237,238] (even if there are some contradictory results [239,240]). Therefore, other tumors are unlikely to contribute to the urinary levels of miR-205.

Conversely, the functional role of miR-31 is extremely complex, as it can act both as an oncogene and tumor suppressor gene in different tumor types [241]. MiR-31 was identified as a circulatory miRNA in serum in carcinomas of the lung, colon, pancreas, breast, and the oral cavity in a large meta-analysis including 1397 cancer patients and 1039 controls [242].

miR-31 expression is downregulated in bladder cancer [243] as well as in invasive urothelial carcinoma of the bladder [244] and in prostate cancer [245]. Thus, again, urinary miR-31 could be a biomarker specific for oncocytoma as its level does not seem to be compromised by secretion into the urine from other urological tumors.

In another work, Von Brandenstein M. et al. also found that miR-15a is a useful urinary biomarker for differentiating malignant renal tumors from benign renal oncocytoma [223]. miR-15a is overexpressed in the urine of RCC patients (*n* = 23: 7 ccRCC, 5 chRCC, 6 papRCC), but is nearly undetectable in the urine of patients with oncocytoma (*n* = 5), other tumors, or urinary tract inflammation [223]. The expression levels of miR-15a are inversely correlated to those of PKC α, a component of the transcription complex in tumors, which is upregulated in benign oncocytomas, but downregulated in RCCs [223]. In renal carcinomas, after nuclear transmigration, PKC α binds directly to pri-miRNA-15a in the nucleus, suppressing its release and the generation of mature miR-15a [223].

The results of Von Brandenstein M. et al. [223] were confirmed in another study in which the authors showed that miR-15a was significantly upregulated in the urine of RCC patients (*n* = 52: 22 ccRCC, 16 papRCC, 14 chRCC) in comparison with that of benign renal tumor patients (*n* = 15: 8 oncocytomas, 2 papillary adenomas, 5 AML) and healthy controls (*n* = 15) (*p* < 0.01), while miR-15a expression in RCCs decreased significantly post-operatively [246]. Pre-operative urine miR-15a levels could discriminate RCCs from benign renal lesions with 98.1% specificity and 100% sensitivity (95% CI 0.9–1.0), with area under the curve (AUC) of the ROC curve equal to 0.955 [246].

It is also clinically important to distinguish early stage small renal masses (SRMs; pT1a, ≤4 cm) from benign lesions. RCC is often detected incidentally as a SRM [247], and up to 30% of surgically treated SRMs are benign on final pathological evaluation [248]. Moreover, RCC-SRMs can either be classified as clinically progressive (grow rapidly and metastasize) or non-progressive. The former requires immediate treatment while the latter, having relatively indolent clinical behavior, can be managed by active surveillance [248,249,250]. It is clinically challenging to predict progression in patients with SRMs, especially for elderly and infirm patients, where the risk of surgery outweighs mortality from SRMs [251].

Di Meo et al., in a cohort of 30 renal oncocytoma (≤4 cm) patients and 26 progressive and 24 non-progressive clear cell RCC-SRM (ccRCC-SRM) patients, identified nine urinary miRNAs (miR-432-5p, miR-532-5p, miR-10a-5p, miR-144-3p, miR-28-3p, miR-326, miR-328-3p, miR-603, miR-93-3p) significantly overexpressed in ccRCC-SRM patients relative to renal oncocytoma patients [252]. MiR-432-5p showed the most significant discriminatory ability between ccRCC-SRM and renal oncocytoma patients (AUC: 0.71, 95% CI: 0.59 to 0.83, *p* = 0.0031) [252]; however, it was still too low to differentiate individual cases of oncocytoma in an everyday clinical setting.

Since ccRCCs exhibit distinct chromosomal aberrations (e.g., whole or partial chromosomal amplification or deletion), genomic alterations may partly explain the differential miRNA expression profiles observed by the authors in renal oncocytoma versus ccRCC-SRMs [252].

Finally, Butz H et al. demonstrated that a combination of urinary miR-126-3p and miR-486-5p was able to differentiate between benign lesions (*n* = 24) and healthy controls (*n* = 33) with an AUC of 0.85 (95% CI, 0.7295–0.9615; *p* < 0.0001) and a sensitivity and specificity of 75% and 87.5%, respectively [253].

They also found other combinations of urinary miRNAs (miR-126-3p–miR-34b-5p; miR-21-5p–miR-34b-5p; miR-126-3p–miR-449a; miR-17-5p–miR-34b-5p; miR-25-3p–miR-34b-5p; miR-34b-5p–miR-1183) able to differentiate benign lesions from healthy controls, but they showed poorer performance (AUC from 0.82 to 0.75) [253]. A miRNA combination of urinary miR-17-5p and miR-25-3p was able to differentiate benign renal tumors from both all ccRCCs considered in the study and from SRMs with an AUC of 0.65 (95% CI, 0.5381–0.7609; *p* = 0.0269; sensitivity = 48.8%; specificity = 87.5%) and 0.68 (95% CI, 0.5456–0.8065; *p* = 0.0191; sensitivity = 52.5%; specificity = 87.5%), respectively [253]. In addition, the ratio of urinary miR-17-5p and miR-21-5p was also able to discriminate benign tumors from SRMs with an AUC of 0.70 (95% CI, 0.5658–0.8300; *p* = 0.0084; sensitivity = 47.5%; specificity = 87.5%) [253].

Again, these discriminatory abilities are still not good enough to differentiate individual cases of oncocytoma in an everyday clinical setting.

Clearly, albeit promising, the research on urinary miRNAs as a new diagnostic tool for oncocytoma patients is still in its infancy. One of the main limitations of the above-described studies was the small sample size. Larger-scale future studies are needed to establish clinical applicability.

Unlike urinary miRNA biomarkers, there are no studies involving plasma/serum miRNAs to specifically diagnose oncocytoma. There are only few studies in which oncocytoma patients were analyzed together with patients with other benign lesions or where the histology of benign lesions was not specified. However, benign lesions, unlike oncocytomas that are clinically detected, such as AML, cystic disease, fibrosis, and glomerulosclerosis, are different from a biological point of view.

In three works, the authors found no difference in the expression levels of the analyzed miRNAs in the serum of patients with RCC or benign lesions or in healthy controls [254,255,256].

Wulfken et al. showed that serum miR-1233 was upregulated in RCC patients with respect to control patients, but its serum level was similar in patients with AML (*n* = 3) or oncocytoma (*n* = 10) and RCC patients (*n* = 84) [254]. Hauser et al. showed that the level of miR-378 was similar in RCC patients (*n* = 117), control subjects (*n* = 123) and patients with benign renal tumors (*n* = 14) [255].

Finally, Heinemann et al. analyzed the small RNA expression profile in the serum of 18 ccRCCs and in eight patients with benign renal tumors using small RNA sequencing [256]. They detected 29 differentially expressed miRNAs and selected three miRNAs (miR-122-5p, miR-193a-5p, and miR-206) among the ones that were strongly expressed and that had not been studied in serum by other researchers for further validation [256]. However, those miRNAs were also already circulating at similar levels in ccRCC patients (*n* = 68) and in patients with benign renal tumors (*n* = 47) in the validation cohort [256].

Conversely, Cheng et al. showed that miR-141 was downregulated, and miR-224, miR-21, and miR-34a were upregulated in the sera of patients with ccRCC (*n* = 12) (*p* < 0.01 for all miRNAs) compared with those of patients with benign kidney lesions (*n* = 12), consistent with their expression in paired tumor tissue samples [257].

Therefore, serum miR-1233, miR-378, miR-122-5p, miR-193a-5p, and miR-206 are unlikely to provide helpful information for detecting benign kidney lesions [254,255,256].

Conversely, serum miR-141, miR-224, miR-21, and miR-34a could be promising [257], but further studies are required to confirm these findings.

Renal oncocytoma is the most prevalent benign lesion of the kidney, and recent evidence suggests that renal AML can be safely diagnosed by imaging [258].

Thus, studies aimed at identifying non-invasive biomarkers specific for the diagnosis of oncocytoma could be highly relevant and should be addressed in the future.

## Figures and Tables

**Table 1:** The Fuhrman grading system [98].

Grade	Criteria
Grade 1	The neoplastic cell nuclei are small and round. The nucleoli are difficult to see even when the cells are examined with a high magnification lens.
Grade 2	The neoplastic cell nuclei are slightly larger and irregularly shaped. Nucleoli are easier to see but only after the cells are examined with a high magnification lens.
Grade 3	The neoplastic cell nuclei are obviously irregular and enlarged. The nucleoli are easy to see even when the cells are examined with a low magnification lens.
Grade 4	The neoplastic cell nuclei are bizarre, extremely irregular and often multilobed. Sarcomatoid and rhabdoid cells are included in this category.

**Table 2:** The WHO/ISUP grading system for ccRCC and papRCC, adapted from [103,104].

Grade	Criteria from the Original Classification for Both Ccrcc and Paprcc	Criteria from the Revised Classification, Tumor Necrosis Integrated, for Ccrcc Only
Grade 1	Tumor cell nucleoli absent or inconspicuous and basophilic at 400× magnification	WHO/ISUP grade 1 WHO/ISUP grade 2, without necrosis
Grade 2	Tumor cell nucleoli conspicuous and eosinophilic at 400× magnification and visible but not prominent at 100× magnification	WHO/ISUP grade 2, necrosis WHO/ISUP grade 3, without necrosis
Grade 3	Tumor cell nucleoli conspicuous and eosinophilic at 100× magnification	WHO/ISUP grade 3 with necrosis WHO/ISUP grade 4 without necrosis
Grade 4	Tumors showing extreme nuclear pleomorphism, tumor giant cells and/or the presence of any proportion of tumor showing sarcomatoid and/or rhabdoid differentiation	WHO/ISUP grade 4 with necrosis Sarcomatoid and/or rhabdoid differentiation

**Table 3:** Histologic and IHC features in the differential diagnosis between eosinophilic renal neoplasms.

Renal Mass	Gross Anatomy	Histology and Architecture	Cytology	CK7	KIT	Vimentin
RO	Solid appearance mahogany brown-tan, brown color 30–50% central scar Less common: hemorrhagic areas (20–27.5%), fat-infiltration, and renal vein invasion, (pseudo-malignant features)	Variable Common: nested, solid, tubular, cystic, tubule-cystic Uncommon: with papillary changes, trabecular, Pseudomalignant: with foci of clear cells, of chRCC-like cells (<5% of tumor extension) or small cells and pseudorosettes, foci of coagulative necrosis (1.8%)	Round-regular nuclei, central and sometimes prominent nucleoli Eosinophilic granular cytoplasm ≤1 mitotic figure	<5% positivity Focal staining of sparse or cluster of cells	Positive	Negative Focal staining of central scar
chRCC	Uncommon central scar Malignant features	Solid Trabecular	Irregular wrinkled, (“raisinoid”) nuclei, nuclear atypia Granular eosinophilic cytoplasm (eosinophilic variant) Granular clear cytoplasm (classic chRCC)	Positive (>5%) Diffuse staining especially (eosinophilic variant > classic chRCC)	Positive	Negative
HOCT	Similar to RO and chRCC	Solid-alveolar	Round nuclei, prominent nucleoli, granular eosinophilic oncocytes with indistinct cytoplasmic margins + chRCC-like cells with slight eosinophilia, irregular nucleus, and distinct cytoplasmic borders Perinuclear halos and binucleated cells	Positive in the majority of cases	Often focal positivity	Positive only focally or negative
HOT	Solid brown single mass	Solid-nested sometimes tubulocystic or trabecular similarly to RO	No significant nuclear atypia if not in few cases, with irregular nuclei and binucleation Prominent and large nucleoli Eosinophilic cells with granular cytoplasm, featuring vacuoles or eosinophilic inclusions (proposed renomination in “sporadic RCC with eosinophilic and vacuolated cytoplasm”) Prominent cytoplasmic membranes Thick-walled vessels	Negative Only single cells stain positive	Positive (up to 64%)	Negative
LOT	Solid, well-circumscribed, single brown mass	Solid, nested Only focally tubular	Regular and round nuclei without significant nuclear atypia indicative of a low-grade, and fine chromatin Polygonal eosinophilic cells, with vacuoles and inclusions	Positive	Negative	Variable From negative to <35% positive
ESC RCC	Defined capsule Solid and macrocystic appearance	Diffuse nested or acinar	Eosinophilic component: eosinophilic cells, with basophilic coarse granules (“stippling”), round-oval nuclei, prominent nucleoli, and only focal nuclear membrane irregularities. Cystic component: cystic trabeculae, the cysts’ lumen is lined with neoplastic epithelium having a hobnail arrangement. Common: presence of histiocytes and lymphocytes	Negative	Negative	Positive
Non-chromophobe RCC	Solid appearance Hemorrhagic areas Uncommon central scar Perinephric fat infiltration, renal vein invasion	Variable (solid; papillary; tubulocystic)	Variable Usually, round to oval irregular nuclei, with nuclear-nucleolar atypia Hobnailing with macronucleoli in tubulocystic RCC WHO/ISUP grading system of nuclear findings used for staging purpose in ccRCCs and papRCC	Variable Minimal in the eosinophilic variant of papRCC	Negative	Positive

**Table 4:** Comparison of US, CT, MRI and SPECT techniques in oncocytoma diagnosis.

	US	CT	MRI	SPECT
Morphology	Well-defined, variable echogenicity, more often hyperechogenic, 20% mild vascularization with CD	Well-defined, mildly hypervascular/hyperdense with respect to renal parechyma	Well-defined, >60% homogeneous signal intensity T1—hypointense signal, but variable findings lead to overlap with RCC T2—hyperintensity, variable	Hypermetabolic masses, can be combined with CT to increase the definition and to obtain tomographic scans
Enhancement, contrast media or radiotracer	85% hyperenhancing, half of them have delayed venous wash-out, no specific microperfusion patterns Spoke-wheel vascular pattern when the central scar is present	Hyperenhancing, delayed wash-out Spoke-wheel pattern if a central scar is present SEI—present but not different with RCC	60% isointense in the dynamic post-gadolinium phase, 40% hypointense in the delayed phase, 80% isointense and 20% hypointense Central scar -T1 hypointensity, T2 hyperintensity, possible delayed enhancement and a spoke-wheel pattern SEI—present but not different from RCC	Hypermetabolic mass, significantly higher values, early and delayed relative uptake versus other RCCs, especially in the delayed phase (>120′)
Additional features		Radiomics—remarkable and promising, especially regarding SEI and radiomic signature, need further validation	DWI—a higher and significantly different ADC from RCC is inconsistently reported Radiomics—remarkable and promising, need further validation	
Diagnostic accuracy	Suboptimal, especially for solid small renal masses, 21–58%	Variable and inconsistent reports in distinguishing RO from RCC	Variable and inconsistent reports in distinguishing RO from RCC	SPECT/CT has 87.5–100% sensitivity, 95.2–96.6% specificity

## Data Availability

Not applicable.
